# *SHROOM3 *is a novel candidate for heterotaxy identified by whole exome sequencing

**DOI:** 10.1186/gb-2011-12-9-r91

**Published:** 2011-09-21

**Authors:** Muhammad Tariq, John W Belmont, Seema Lalani, Teresa Smolarek, Stephanie M Ware

**Affiliations:** 1 Division of Molecular Cardiovascular Biology, Cincinnati Children's Hospital Medical Center, 3333 Burnet Avenue, Cincinnati, OH 45229, USA; 2 Department of Molecular and Human Genetics, Baylor College of Medicine, One Baylor Plaza, Houston, TX 77030, USA; 3 Division of Human Genetics, Cincinnati Children's Hospital Medical Center, 3333 Burnet Avenue, Cincinnati, OH 45229, USA

## Abstract

**Background:**

Heterotaxy-spectrum cardiovascular disorders are challenging for traditional genetic analyses because of clinical and genetic heterogeneity, variable expressivity, and non-penetrance. In this study, high-resolution SNP genotyping and exon-targeted array comparative genomic hybridization platforms were coupled to whole-exome sequencing to identify a novel disease candidate gene.

**Results:**

SNP genotyping identified absence-of-heterozygosity regions in the heterotaxy proband on chromosomes 1, 4, 7, 13, 15, 18, consistent with parental consanguinity. Subsequently, whole-exome sequencing of the proband identified 26,065 coding variants, including 18 non-synonymous homozygous changes not present in dbSNP132 or 1000 Genomes. Of these 18, only 4 - one each in *CXCL2*, *SHROOM3*, *CTSO*, *RXFP1 *- were mapped to the absence-of-heterozygosity regions, each of which was flanked by more than 50 homozygous SNPs, confirming recessive segregation of mutant alleles. Sanger sequencing confirmed the *SHROOM3 *homozygous missense mutation and it was predicted as pathogenic by four bioinformatic tools. *SHROOM3 *has been identified as a central regulator of morphogenetic cell shape changes necessary for organogenesis and can physically bind ROCK2, a rho kinase protein required for left-right patterning. Screening 96 sporadic heterotaxy patients identified four additional patients with rare variants in *SHROOM3*.

**Conclusions:**

Using whole exome sequencing, we identify a recessive missense mutation in *SHROOM3 *associated with heterotaxy syndrome and identify rare variants in subsequent screening of a heterotaxy cohort, suggesting *SHROOM3 *as a novel target for the control of left-right patterning. This study reveals the value of SNP genotyping coupled with high-throughput sequencing for identification of high yield candidates for rare disorders with genetic and phenotypic heterogeneity.

## Background

Congenital heart disease (CHD) is the most common major birth defect, affecting an estimated 1 in 130 live births [1]. However, the underlying genetic causes are not identified in the vast majority of cases [2,3]. Of these, approximately 25% are syndromic while approximately 75% are isolated. Heterotaxy is a severe form of CHD, a multiple congenital anomaly syndrome resulting from abnormalities of the proper specification of left-right (LR) asymmetry during embryonic development, and can lead to malformation of any organ that is asymmetric along the LR axis. Heterotaxy is classically associated with heart malformations, anomalies of the visceral organs such as gut malrotation, abnormalities of spleen position or number, and situs anomalies of the liver and/or stomach. In addition, inappropriate retention of symmetric embryonic structures (for example, persistent left superior vena cava), or loss of normal asymmetry (for example, right atrial isomerism) are clues to an underlying disorder of laterality [4,5].

Heterotaxy is the most highly heritable cardiovascular malformation [6]. However, the majority of heterotaxy cases are considered idiopathic and their genetic basis remains unknown. To date, point mutations in more than 15 genes have been identified in humans with heterotaxy or heterotaxy-spectrum CHD. Although their prevalence is not known with certainty, they most likely account for approximately 15% of heterotaxy spectrum disorders [4,7-9]. Human X-linked heterotaxy is caused by loss of function mutations in *ZIC3*, and accounts for less than 5% of sporadic heterotaxy cases [9]. Thus, despite the strong genetic contribution to heterotaxy, the majority of cases remain unexplained and this indicates the need for utilization of novel genomic approaches to identify genetic causes of these heritable disorders.

LR patterning is a very important feature of early embryonic development. The blueprint for the left and right axes is established prior to organogenesis and is followed by transmission of positional information to the developing organs. Animal models have been critical for identifying key signaling pathways necessary for the initiation and maintenance of LR development. Asymmetric expression of Nodal, a transforming growth factor beta ligand, was identified as an early molecular marker of LR patterning that is conserved across species [10-12]. Genes in the Nodal signaling pathway account for the majority of genes currently known to cause human heterotaxy. However, the phenotypic variability of heterotaxy and frequent sporadic inheritance pattern have been challenging for studies using traditional genetic approaches. Although functional analyses of rare variants in the Nodal pathway have been performed that confirm their deleterious nature, in many cases these variants are inherited from unaffected parents, suggesting that they function as susceptibility alleles in the context of the whole pathway [7,8].

More recent studies have focused on pathways upstream of Nodal signaling, including ion channels and electrochemical gradients [13-15], ciliogenesis and intraflagellar transport [16], planar cell polarity (Dvl2/3, Nkd1) [17,18] and convergence extension (Vangl1/2, Rock2) [19,20], and non-transforming growth factor beta pathway members that interact with the Nodal signaling pathway (for example, Ttrap, Geminin, Cited2) [21-23]. Relevant to the current study, we recently identified a rare copy number variant containing *ROCK2 *in a patient with heterotaxy and showed that its knockdown in *Xenopus *causes laterality defects [24]. Similar laterality defects were identified separately with knockdown of *Rock2b *in zebrafish [20]. The emergence of additional pathways regulating LR development has led to new candidates for further evaluation. Given the mutational spectrum of heterotaxy, we hypothesize that whole-exome approaches will be useful for the identification of novel candidates and essential for understanding the contribution of susceptibility alleles to disease penetrance.

Very recently, whole-exome analysis has been used successfully to identify the causative genes for many rare disorders in affected families with small pedigrees and even in singlet inherited cases or unrelated sporadic cases [25-29]. Nevertheless, one of the challenges of whole-exome sequencing is the interpretation of the large number of variants identified. Homozygosity mapping is one approach that is useful for delineating regions of interest. A combined approach of homozygosity mapping coupled with partial or whole-exome analysis has been used successfully in identification of disease-causing genes in recessive conditions focusing on variants within specific homozygous regions of the genome [30-32]. Here we use SNP genotyping coupled to a whole-exome sequencing strategy to identify a novel candidate for heterotaxy in a patient with a complex heterotaxy syndrome phenotype. We further evaluate *SHROOM3 *in an additional 96 patients from our heterotaxy cohort and identify four rare variants, two of which are predicted to be pathogenic.

## Results

### Phenotypic evaluation

Previously we presented a classification scheme for heterotaxy in which patients were assigned to categories, including syndromic heterotaxy, classic heterotaxy, or heterotaxy spectrum CHD [9]. Using these classifications, patient LAT1180 was given a diagnosis of a novel complex heterotaxy syndrome based on CHD, visceral, and other associated anomalies. Clinical features include dextrocardia, L-transposition of the great arteries, abdominal situs inversus, bilateral keratoconus, and sensorineural hearing loss (Table 1). The parents of this female proband are first cousins, suggesting the possibility of an autosomal recessive condition.

**Table 1:** Clinical findings in LAT1180

Clinical findings in LAT1180
Dextrocardia
L-Transposition of the great arteries
Pulmonic stenosis
Abdominal situs inversus
Bilateral keratoconus
Sensorineural hearing loss
Multiple nevi
Malignant melanoma

### Chromosome microarray analysis

**Figure 1:**
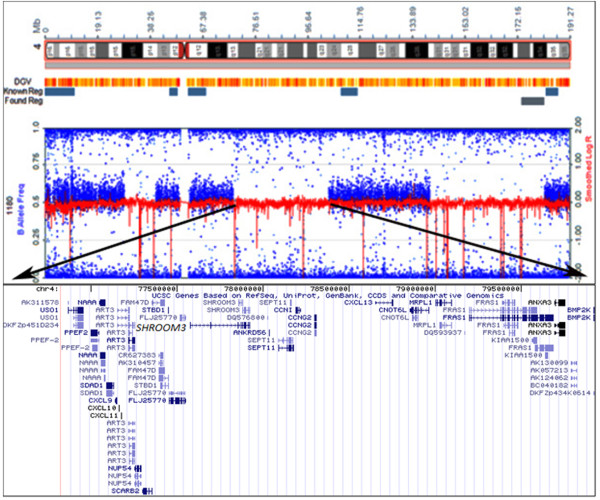
**Screenshot from KaryoStudio software showing ideogram of chromosome 4 and absence-of-heterozygosity regions in LAT1180**. One of these regions, highlighted by arrows, contains *SHROOM3*. A partial gene list from the region is shown. DGV, Database of Genomic Variants.

LAT1180 was assessed for submicroscopic chromosomal abnormalities using an Illumina genome-wide SNP array as well as exon-targeted array comparative genomic hybridization (aCGH). Copy number variation (CNV) analysis did not identify potential disease-causing chromosomal deletions/duplications. However, several absence-of-heterozygosity regions (homozygous runs) were identified via SNP genotyping analysis (Table 2 and Figure 1), consistent with the known consanguinity in the pedigree. These regions have an overwhelming probability to carry disease mutations in inbred families [33].

**Table 2:** Major absence-of-heterozygosity regions identified in LAT1180 using SNP array

Chromosome	Start (bp)	Stop (bp)	Length (bp)	Cytobands	Number of markers	Genes in region
1	186823646	192715568	5891922	q31.1-q31.3	1,533	13
4	69717060	89279933	33212166	q13.2-q24	> 8,000	> 200
4	146672223	182010642	35838420	q31.21-q34.3	8,626	> 100
7	40952323	47059534	6107211	p14.1-p12.3	2,324	47
13	40907456	47064783	6157327	q14.11-q14.2	2,461	35
15	46957310	51984619	5027309	q21.1-q21.3	1,792	41
18	22763465	33898685	11135220	q11.2-q12.2	4,107	45

### Exome analysis

**Figure 2:**
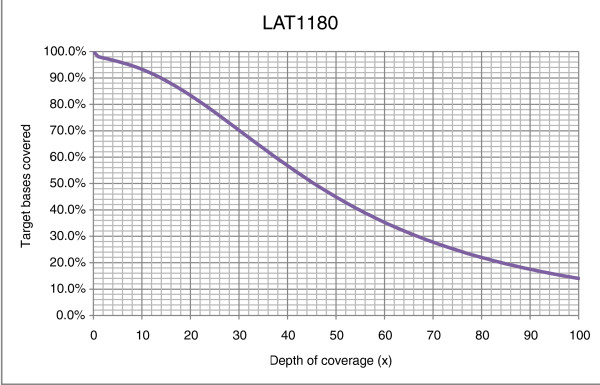
**Comparsion of depth of coverage (x-axis) and percentage of target bases covered (y-axis) from exome analysis of LAT1180**.

Following SNP microarray and aCGH, the exome (36.5 Mb of total genomic sequence) of LAT1180 was sequenced to a mean coverage of 56-fold. A total of 5.71 Gb of sequence data was generated, with 53.9% of bases mapping to the consensus coding sequence exome (accession number [NCBI: SRP007801]) [34]. On average, 93.3% of the exome was covered at 10× coverage (Table 3 and Figure 2), and 70,812 variants were identified, including 26,065 coding changes (Table 4). Overall, our filtering strategy (Materials and methods) identified 18 homozygous missense changes with a total of 4 coding changes occurring within the previously identified absence-of-heterozygosity regions (Table 2 and Figure 1). These included one variant each in *CXCL2 *(p.T39A; chr4:74,964,625), *SHROOM3 *(p.G60V; chr4:77,476,772), *CTSO *(p.Q122E; chr4:156,863,489), and *RXFP1 *(p.T235I; chr4:159,538,306).

**Table 3:** Exome statistics for LAT1180

Total amount of raw data generated (Gb)	5.71
Sequencing read length (bp)	50
Total reads generated (million pairs)	57.091
Reads aligning to human reference genome hg19 (million pairs)	47.640
Usable data for alignment (Gb)	4.76
Reads aligned to human reference genome hg19	83.4%
Bases aligning to human exome (targets)	53.9%
Total bases aligning to exome (Gb)	2.57
Mean depth of coverage of targets	56
Maximum depth of coverage of targets	2434
Minimum depth of coverage of targets	0
Average depth of coverage	58
Bases covered at depth of ≥ 1×	98.1%
Bases covered at depth of ≥ 5×	96.3%
Bases covered at depth of ≥ 10×	93.3%

**Table 4:** Exome sequencing and filtering strategy in LAT1180¶

Total variants identified	70,812
Total coding variants identified	26,065
Total dbSNP132 variants	63,728
Total changes not present in dbSNP132 database	7,084
Coding changes	4,351
Homozygous missense changes	62
Homozygous missense changes not present in 1000 Genomes data	36
Homozygous missense changes on chromosomes 1, 4, 7, 13, 15, 18	18
Homozygous missense changes within absence-of-heterozygosity	4

¶An autosomal recessive inheritance model was assumed.

Previously, we developed an approach for prioritization of candidate genes for heterotaxy spectrum cardiovascular malformations and laterality disorders based on developmental expression and gene function [24]. In addition, we have developed a network biology analysis appropriate for evaluation of candidates relative to potential interactions with known genetic pathways for heterotaxy, LR patterning, and ciliopathies in animal models and humans (manuscript in preparation). Using these approaches, three of the genes, *CXCL2*, *CTSO*, and *RXFP1*, are considered unlikely candidates. *CXCL2 *is an inducible chemokine important for chemotaxis, immune response, and inflammatory response. Targeted deletion of *Cxcl2 *in mice does not cause congenital anomalies but does result in poor wound healing and increased susceptibility to infection [35]. CTSO, a cysteine proteinase, is a proteolytic enzyme that is a member of the papain superfamily involved in cellular protein degradation and turnover. It is expressed ubiquitously postnatally and in the brain prenatally. *RFXP1 *(also known as *LRG7*) is a G-protein coupled receptor to which the ligand relaxin binds. It is expressed ubiquitously with the exception of the spleen. Mouse Genome Informatics shows that homozygous deletion of *Rfxp1 *leads to males with reduced fertility and females unable to nurse due to impaired nipple development. In contrast, *SHROOM3 *is considered a very strong candidate based on its known expression and function, including its known role in gut looping and its ability to bind ROCK2.

**Figure 3:**
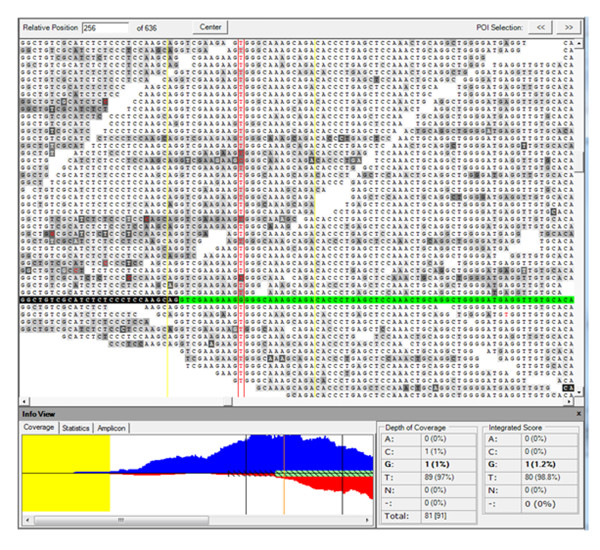
**Alignment of exome high-throughput sequencing data showing *SHROOM3 *gene mutation c.179G > T bordered by red vertical lines**. The *SHROOM3 *sequence (RefSeq ID: NG_028077.1) is shown by a single row containing both exonic (green) and intronic (black) areas. The lower left corner of the figure shows the sequencing depth of coverage of exonic sequences (protein-coding) as a green bar. The blue area shows the forward strand sequencing depth while red shows reverse strand sequencing depth. Yellow represents the non-genic and non-targeted sequences of the genome. The mutation call rate is 99% (89 reads with T versus 1 read with C at c.179 of the *SHROOM3 *gene).

**Figure 4:**
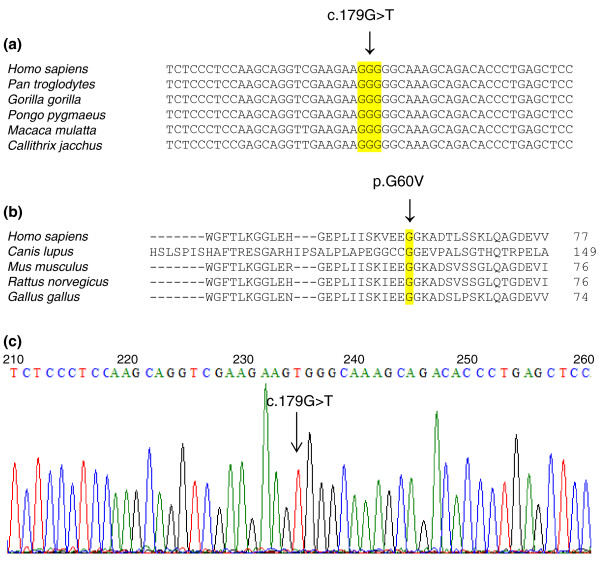
**Cross-species analysis and *SHROOM3 *mutation**. **(a) **Partial nucleotide sequence of *SHROOM3 *from different species showing conserved codon for glycine at amino acid position 60 and mutated nucleotide G shown by an arrow. **(b) **Partial amino acid sequence of SHROOM3 proteins from different species highlighting conservation of glycine. **(c) **Partial *SHROOM3 *chromatogram from LAT1180 DNA showing homozygous mutation G > T by an arrow.

Further analysis of the *SHROOM3 *gene confirmed a homozygous missense mutation (Table 4 and Figure 3) in a homozygous run on chromosome 4. These data support the recessive segregation of the variant with the phenotype. This mutation was confirmed by Sanger sequencing (Figure 4c) and was predicted to create a cryptic splice acceptor site, which may cause loss of exon 2 of the gene.

### Pathogenicity prediction

**Figure 5:**
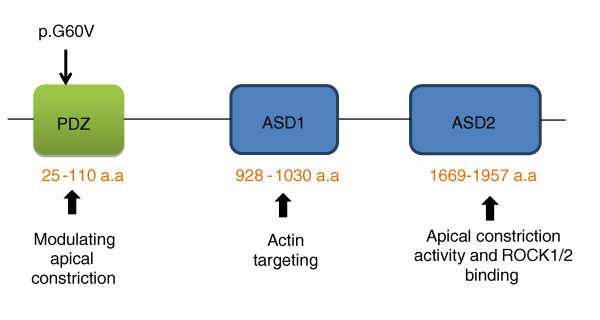
**Representative structure of SHROOM3 showing three main functional protein domains: PDZ, ASD1, and ASD2**. a.a, amino acid; ASD, Apx/Shrm domain; Dlg1, *Drosophila *disc large tumor suppressor; PDZ, post-synaptic density protein (PSD95); zo-1, zonula occludens-1 protein.

The homozygous mutation p.G60V in *SHROOM3 *was predicted to be pathogenic using the bioinformatic programs Polyphen-2 [36], PANTHER [37], Mutation Taster [38] and SIFT [39]. Glycine at position 60 of SHROOM3 as well as its respective triplet codon (GGG) in the gene are evolutionarily conserved across species, suggesting an important role of this residue in protein function (Figure 4a, b). Mutation Taster [38] predicted loss of the PDZ domain (25 to 110 amino acids) and probable loss of remaining regions of SHROOM3 protein due to the cryptic splicing effect of the c.179G > T mutation in the gene (Figure 5). Variants in *CTSO*, *RFXP1*, and *CXCL2 *were predicted to be benign by more than two of the above bioinformatic programs.

### Mutation screening

**Figure 6:**
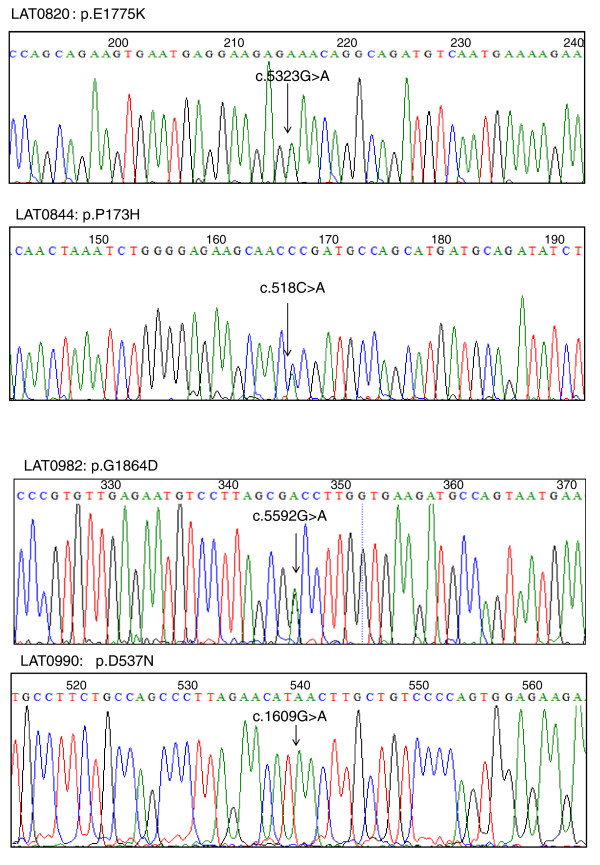
**Non-synonymous rare variants identified in *SHROOM3 *mutation screening in heterotaxy patients**. Partial *SHROOM3 *chromatogram showing homozygous rare variants in samples from LAT0820 and LAT0990 and heterozygous variants in LAT0844 and LAT0982. Arrows indicate position of nucleotide changes.

*SHROOM3 *was analyzed in 96 sporadic heterotaxy patients with unknown genetic etiology for their disease using PCR amplification followed by Sanger sequencing. Four nonsynonymous nucleotide changes were identified (Table 5 and Figure 6) that were not present in the HapMap or 1000 Genomes databases, indicating they are rare variants. Each variant was analyzed using PolyPhen, SIFT, and PANTHER. Both homozygous variants p.D537N and p.E1775K were predicted to be benign by all programs, whereas the heterozygous variants p.P173H and p.G1864D were identified as damaging by all programs.

**Table 5:** Rare variants in *SHROOM3*

Patient ID	Amino acid	Predicted pathogenicity	Allele	hg19 coordinates
LAT0820	p.E1775K	- - -	Homozygous	chr4: 77,680,822
LAT0844	p.P173H	+ + +	Heterozygous	chr4:77,652,019
LAT0982	p.G1864D	+ + +	Heterozygous	chr4:77,692,019
LAT0990	p.D537N	- - -	Homozygous	chr4: 77,660,935
LAT1180	p.G60V	+ + +	Homozygous	chr4:77,476,772

Predicted pathogenicity results are presented for PolyPhen, SIFT, and PANTHER analysis. +, probably damaging or damaging (deleterious); -, benign.

## Discussion

In the present study, we investigated a proband, LAT1180, from a consanguineous pedigree with a novel form of heterotaxy syndrome using microarray-based CNV analysis and whole-exome sequencing. Our initial genetic analysis using two microarray-based platforms (Illumina SNP genotyping and exon-targeted Agilent aCGH) failed to identify any potential structural mutation. However, we observed homozygous regions (absence-of-heterozygosity) from SNP genotyping data, suggesting that homozygous point mutations or small insertion/deletion events within these regions could be disease associated. Subsequently, whole-exome analysis resulted in the identification of a novel homozygous missense mutation in the *SHROOM3 *gene on chromosome 4. Additional sequencing in a cohort of 96 heterotaxy patients identified two additional patients with homozygous variants and two patients with heterozygous variants. Although *in vivo *loss of function analyses have demonstrated the importance of SHROOM3 for proper cardiac and gut patterning, specific testing of the variants identified herein will be useful to further establish pathogenicity and the most common mode of inheritance. This study demonstrates the usefulness of high-throughput sequencing and SNP genotyping to identify important candidates in disorders characterized by genetic and phenotypic heterogeneity.

*SHROOM3 *encodes a cytoskeletal protein of 1,996 residues that is composed of 3 main domains with distinct functions (Figure 5). SHROOM3, an actin binding protein, is responsible for early cell shape during morphogenesis through a myosin II-dependent pathway. It is essential for neural tube closure in mouse, *Xenopus*, and chick [40-42]. Early studies in model species showed that Shroom3 plays an important role in the morphogenesis of epithelial sheets, such as gut epithelium, lens placode invagination, and also cardiac development [43,44]. Recent data indicate an important role for Shroom3 in proper gut rotation [45]. Interestingly, gut malrotation is a common feature of heterotaxy and is consistent with a laterality disorder. In *Xenopus*, Shroom3 is expressed in the myocardium and is necessary for cellular morphogenesis in the early heart as well as normal cardiac tube formation with disruption of cardiac looping (Thomas Drysdale, personal communication, manuscript in revision). Downstream effector proteins of Shroom3 include Mena, myosin II, Rap1 GTPase and Rho Kinases [40-42,44,46].

Shroom3 may play an important role in LR development acting downstream of Pitx2. Pitx2 is an important transcription factor in the generation of LR patterning in *Xenopus*, zebrafish, and mice [47-49]. Recently it was shown that Pitx2 can directly activate expression of *Shroom3 *and ultimately chiral gut looping in *Xenopus *[43]. Gut looping morphogenesis in *Xenopus *is most likely driven by cell shape changes in gut epithelium [50]. The identification of Shroom3 as a downstream effector fills an important gap in understanding how positional information is transferred into morphogenetic movements during organogenesis. The presence of a Pitx2 binding-sites upstream of mouse *Shroom3 *combined with the similar gut looping phenotypes of mouse *Pitx2 *and *Shroom3 *mutants supports the interactive mechanism for these two proteins [41,43,51].

**Figure 7:**
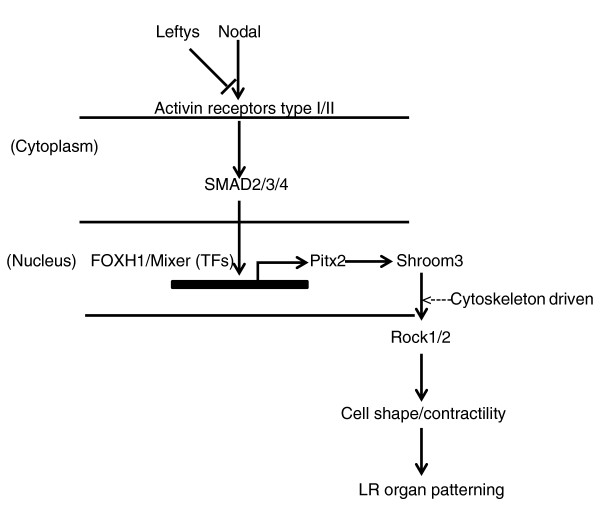
**Proposed model for Shroom3 involvement in LR patterning**. Flow diagram illustrating key interactions in early embryonic LR development. Nodal is expressed asymmetrically at the left of the node (mouse), gastrocoel roof plate (*Xenopus*) or Kuppfer's vesicle (zebrafish), followed by asymmetric Nodal expression in the left lateral plate mesoderm. Pitx proteins bind the *Shroom3 *promoter to activate expression. Studies from animal models also suggest a role of cytoskeleton-driven polarity in LR asymmetry establishment. LR, left-right; TF, transcription factors.

Studies from snails, frogs and mice suggest cell-shape/arrangement regulation and cytoskeleton-driven polarity is initiated early during development, establishing LR asymmetry [19,52-55]. Recent data from our lab and others demonstrated that rho kinase (ROCK2), a downstream effector protein of SHROOM3, is required for LR and anteroposterior patterning in humans, *Xenopus *and zebrafish [20,24]. In animal models, either overexpression or loss of function may cause similar phenotypes. These results led us to suggest that this pathway (Figure 7), which is a central regulator of morphogenetic cell shape changes, may be a novel target for the control of LR patterning. Sequencing of these newly identified genes downstream of the canonical Nodal signal transduction pathway will be necessary to determine their importance for causing heterotaxy in a larger number of patients. We predict whole-exome sequencing will become an important modality for the identification of novel disease-causing heterotaxy genes, candidate genes, and disease-associated rare variants important for disease susceptibility.

## Conclusions

*SHROOM3 *is a novel candidate for heterotaxy-spectrum cardiovascular malformations. This study highlights the importance of microarray-based SNP/CNV genotyping followed by exome sequencing for identification of novel candidates. This approach can be useful for rare disorders that have been challenging to analyze with traditional genetic approaches due to small numbers, significant clinical and genetic heterogeneity, and/or multifactorial inheritance.

## Materials and methods

### Subjects

DNA of proband LAT1180 was extracted from whole peripheral blood leukocytes following a standard protocol. Screening of *SHROOM3 *was performed using DNA samples from 96 additional sporadic heterotaxy patients. The heterotaxy cohort has been reported previously [7,9]. DNA samples with previous positive genetic testing results were not used in the current study. This study was approved by the Institutional Review Boards at the Baylor College of Medicine and Cincinnati Children's Hospital Medical Center (CCHMC). Written informed consent for participation in this study as well as publication of clinical data of the proband was obtained. All the methods applied in this study conformed to the Declaration of Helsinki (1964) of the World Medical Association concerning human material/data and experimentation [56] and ethical approval was granted by the ethics committee of the Baylor College of Medicine and CCHMC.

### SNP genotyping

Genome-wide SNP genotyping was performed using an Illumina HumanOmni-Quad Infinium HD BeadChip. The chip contains 1,140,419 SNP markers with an average call frequency of > 99% and is unbiased to coding and noncoding regions of the genome. CNV analysis was performed using KaryoStudio Software (Illumina Inc.).

### Array comparative genomic hybridization

The custom exon-targeted aCGH array was designed by Baylor Medical Genetics Laboratories [57] and manufactured by Agilent Technology (Santa Clara, CA, USA). The array contains 180,000 oligos covering 24,319 exons (4.2/exon). Data (105 k) were normalized using the Agilent Feature Extraction software. CNVs were detected by intensities of differentially labeled test DNA samples and LAT1180 DNA samples hybridized to Agilent array containing probes (probe-based). Results were interpreted by an experienced cytogeneticist at the Baylor College of Medicine. The Database of Genomic Variants [58] and in-house cytogenetic databases from the Baylor College of Medicine and CCHMC were used as control datasets for CNV analysis.

### Exome sequencing

Genomic DNA (3 μg) from proband LAT1180 was fragmented and enriched for human exonic sequences with the NimbleGen SeqCap EZ Human Exome v2.0 Library (2.1 million DNA probes). A total of approximately 30,000 consensus coding sequence genes (approximately 300,000 exons, total size 36.5 Mb) are targeted by this capture, which contains probes covering a total of 44.1 Mb. The resulting exome library of the proband was sequenced with 50 bp paired-end reads using Illumina GAII (v2 Chemistry). Data are archived at the NCBI Sequence Read Archive (SRA) under an NCBI accession number [NCBI: SRP007801] [34]. All sequence reads were mapped to the reference human genome (UCSC hg 19) using the Illumina Pipeline software version 1.5 featuring a gapped aligner (ELAND v2). Variant identification was performed using locally developed software 'SeqMate' (submitted for publication). The tool combines the aligned reads with the reference sequence and computes a distribution of call quality at each aligned base position, which serves as the basis for variant calling. Variants are reported based on a configurable formula using the following additional parameters: depth of coverage, proportion of each base at a given position and number of different reads showing a sequence variation. The minimum number of high quality bases to establish coverage at any position was arbitrarily set at 10. Any sequence position with a non-reference base observed more than 75% of the time was called a homozygous variant. Any sequence position with a non-reference base observed between 25% and 75% of the time was called a heterozygous variant. Amino acid changes were identified by comparison to the UCSC RefSeq database track. A local realignment tool was used to minimize the errors in SNP calling due to indels. A series of filtering strategies (dbSNP132, 1000 Genomes project (May 2010)) were applied to reduce the number of variants and to identify the potential pathogenic mutations causing the disease phenotype.

### Mutation screening and validation

Primers were designed to cover exonic regions containing potential variants of *SHROOM3 *and *UGT2A1 *genes in LAT1180. For screening additional heterotaxy patients, primers were designed to include all exons and splice junctions of *SHROOM3 *(primer sequences are available upon request). A homozygous nonsense variant (p.Y192X) was confirmed in the *UGT2A1 *gene within the same homozygous region on chromosome 4 but was later excluded because of its presence in the 1000 Genomes project data. PCR products were sequenced using BigDye Terminator and an ABI 3730XL DNA Analyzer. Sequence analysis was performed via Bioedit Sequence Alignment Editor, version 6.0.7 [59]. All positive findings were confirmed in a separate experiment using the original genomic DNA sample as template for new amplification and bi-directional sequencing reactions.

## Abbreviations

aCGH: array comparative genomic hybridization; bp: base pair; CHD: congenital heart disease; CNV: copy number variation; Gb: giga-base pair; LR: left-right; Mb: mega-base pair; SNP: single nucleotide polymorphism.

## Authors' contributions

MT performed experiments, bioinformatics/mutational analysis and Sanger validation and wrote the manuscript. JWB performed clinical diagnosis. SMW performed clinical diagnosis, designed the project, received funding and wrote the manuscript. SL and TS evaluated and interpreted SNP microarray and aCGH data. All authors read and approved the final manuscript for publication.
